# Supplementary material for: Adulthood depletion of Integrator extends lifespan and healthspan via defective pre-mRNA processing
Source: bioRxiv. 2026 Apr 21:2026.04.18.719358. Preprint. [Version 1] doi: 10.64898/2026.04.18.719358 (PMC13131478; doi:10.64898/2026.04.18.719358)
Supplement: 1 [file NIHPP2026.04.18.719358V1-supplement-1.pdf]

bioRxiv preprint doi: https://doi.org/10.64898/2026.04.18.719358; this version posted April 21, 2026. The copyright holder for this preprint (which was not certified by peer review) is the author/funder, who has granted bioRxiv a license to display the preprint in perpetuity. It is made available under aCC-BY 4.0 International license.

## Supplementary figures and legends:

bioRxiv preprint doi: https://doi.org/10.64898/2026.04.18.719358; this version posted April 21, 2026. The copyright holder for this preprint (which was not certified by peer review) is the author/funder, who has granted bioRxiv a license to display the preprint in perpetuity. It is made available under aCC-BY 4.0 International license.

## Supplemental figure 1.

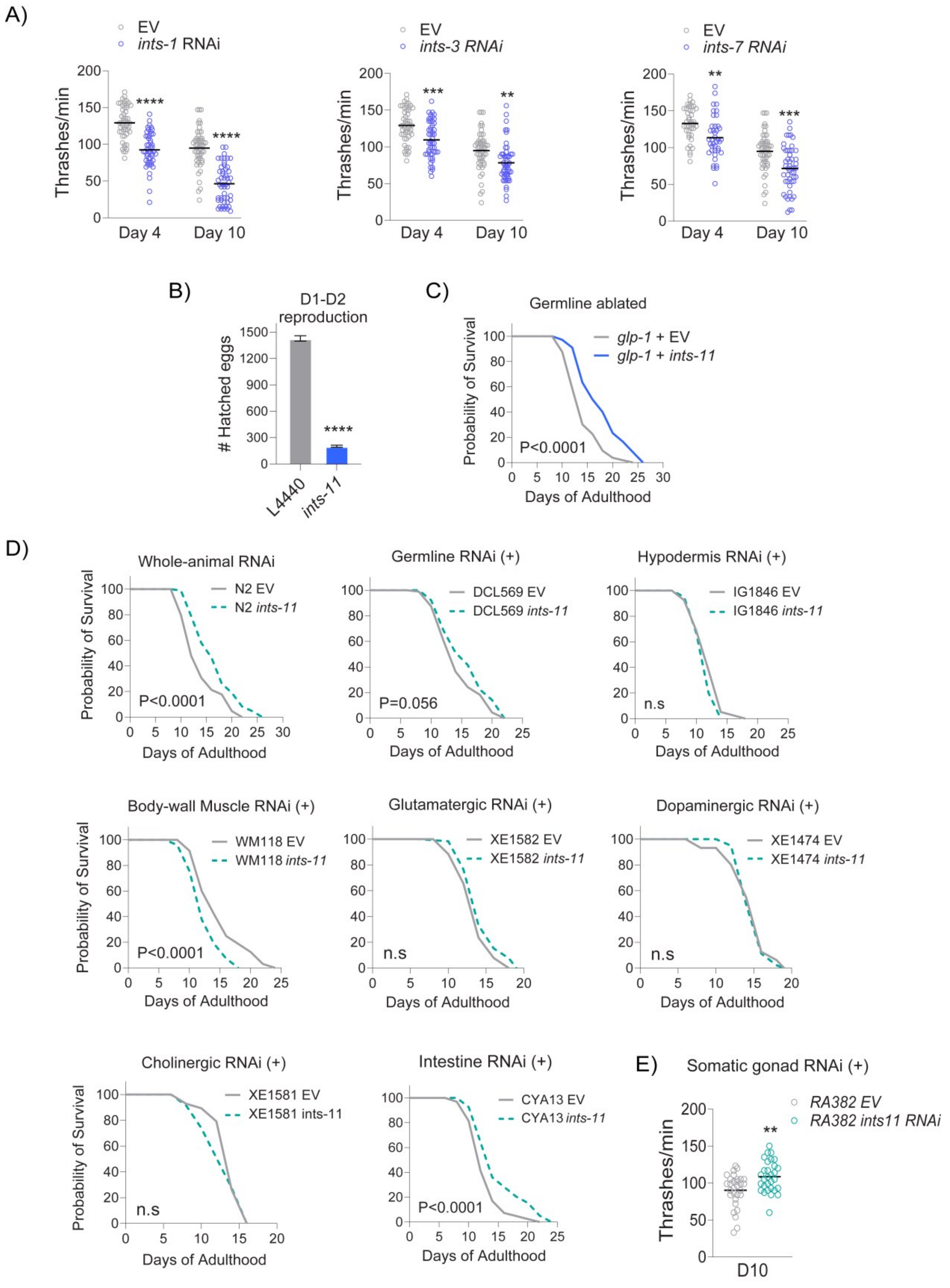

bioRxiv preprint doi: https://doi.org/10.64898/2026.04.18.719358; this version posted April 21, 2026. The copyright holder for this preprint (which was not certified by peer review) is the author/funder, who has granted bioRxiv a license to display the preprint in perpetuity. It is made available under aCC-BY 4.0 International license.

**Supplemental figure 1. A)** Thrashing performance in day-4 and day-10 animals showing impaired healthspan from adulthood depletion of *ints-1, ints-3* and *ints-7* Integrator subunits. **B)** Egg hatching assay from 5 combined representative individuals fed either empty vector or *ints-11* dsRNA from L4 stage. **C)** RNAi against *ints-11* within sterile *glp-1(e2141)* mutants extends lifespan. **D)** Tissue-specific RNAi screen for *ints-11*. **E)** Thrashing rates are enhanced from *ints-11* RNAi within the somatic gonad. Thrashing assays are from 2 independent repeats containing ~50 individuals. Lifespans are biological repeats from ~200 individuals.

bioRxiv preprint doi: https://doi.org/10.64898/2026.04.18.719358; this version posted April 21, 2026. The copyright holder for this preprint (which was not certified by peer review) is the author/funder, who has granted bioRxiv a license to display the preprint in perpetuity. It is made available under aCC-BY 4.0 International license.

**Supplemental figure 2.**

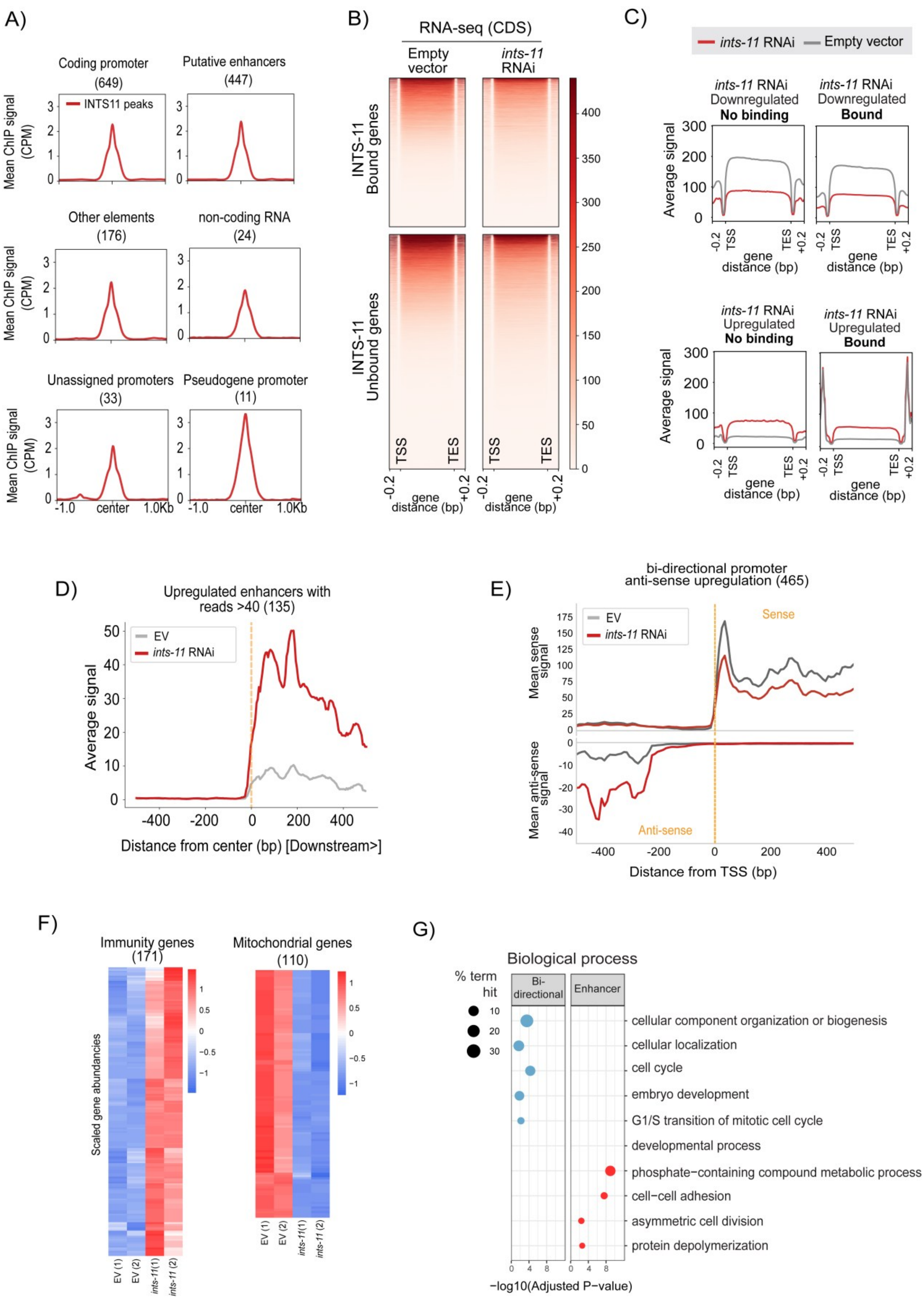

bioRxiv preprint doi: https://doi.org/10.64898/2026.04.18.719358; this version posted April 21, 2026. The copyright holder for this preprint (which was not certified by peer review) is the author/funder, who has granted bioRxiv a license to display the preprint in perpetuity. It is made available under aCC-BY 4.0 International license.

**Supplemental figure 2. A)** Mean chIP-centred peak intensity plots across multiple regulatory transcriptional loci. **B)** Heatmap comparing RNA-seq expression coverage across coding domains of INTS-11 bound versus unbound genes in empty vector (left) and *ints-11* RNAi-fed (right) animals. **C)** Metagene plots across coding domains of INTS11 bound versus unbound genes, but stratified into subsets identified as either differentially up/downregulated within RNA-seq data. **D)** Metagene plot for upregulated enhancer loci transcription for genes with moderate (>40 average reads) RNA-seq expression coverage. **E)** RNA-seq metagene plot for bidirectional promoter antisense-upregulated protein-coding genes. **F)** Scaled heatmaps of mitochondria- and immune-related genes from differentially expressed RNA-seq data. **G)** Gene ontology enrichment of genes associated with upregulated enhancer (left panel) and bidirectional promoter (right panel).

bioRxiv preprint doi: https://doi.org/10.64898/2026.04.18.719358; this version posted April 21, 2026. The copyright holder for this preprint (which was not certified by peer review) is the author/funder, who has granted bioRxiv a license to display the preprint in perpetuity. It is made available under aCC-BY 4.0 International license.

**Supplemental figure 3.**

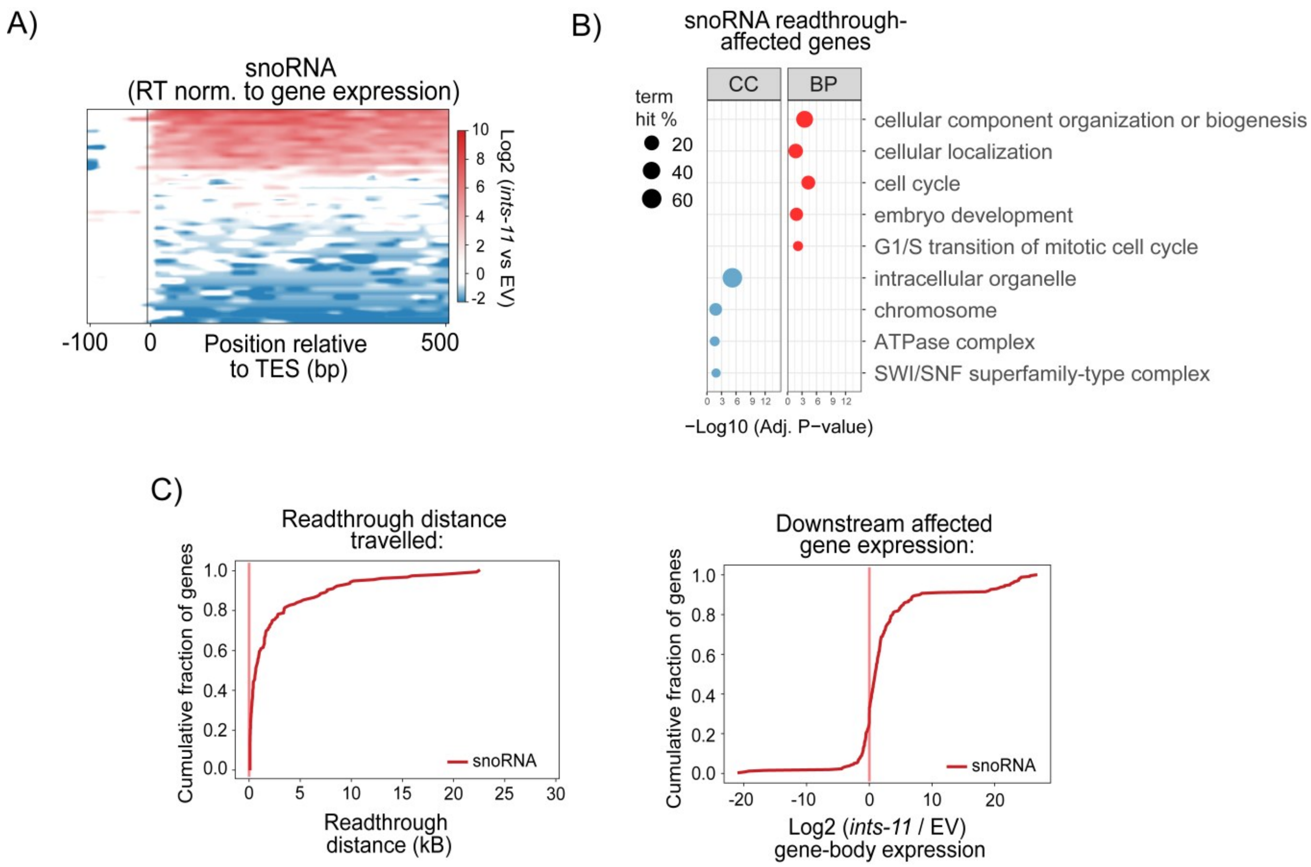

bioRxiv preprint doi: https://doi.org/10.64898/2026.04.18.719358; this version posted April 21, 2026. The copyright holder for this preprint (which was not certified by peer review) is the author/funder, who has granted bioRxiv a license to display the preprint in perpetuity. It is made available under aCC-BY 4.0 International license.

**Supplemental figure 3. A)** Heatmap of pol II readthrough events at snoRNA TES loci. **B)** Gene ontology enrichment analysis of snoRNA readthrough-affected genes. **C)** Cumulative distribution plot of distance travelled from snoRNA readthrough genes (left), and gene expression of downstream affected genes residing within snoRNA readthrough windows (right).

bioRxiv preprint doi: https://doi.org/10.64898/2026.04.18.719358; this version posted April 21, 2026. The copyright holder for this preprint (which was not certified by peer review) is the author/funder, who has granted bioRxiv a license to display the preprint in perpetuity. It is made available under aCC-BY 4.0 International license.

# Supplemental figure 4.

A)

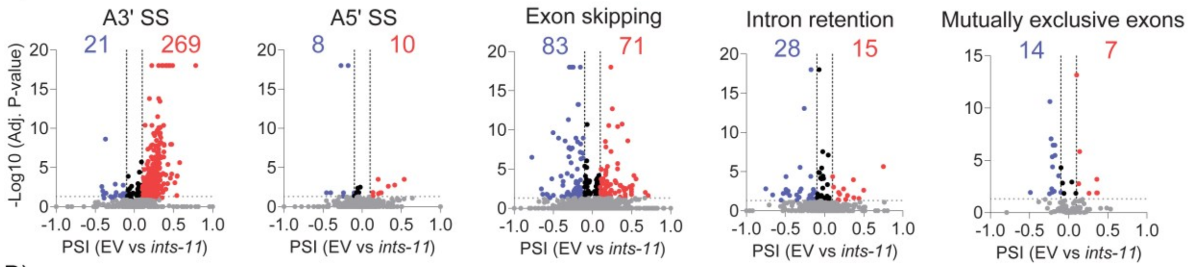


B)

| Splicing type | Number of events | P-adj <0.05 | P-adj & FC |
|---|---|---|---|
| A3SS | 862 | 334 | 290 |
| A5SS | 454 | 23 | 18 |
| MXE | 312 | 26 | 21 |
| RI | 599 | 67 | 43 |
| SE | 4,158 | 208 | 154 |
| | 11,460 | 658 | 526 |

C)

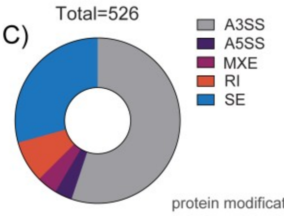


D)

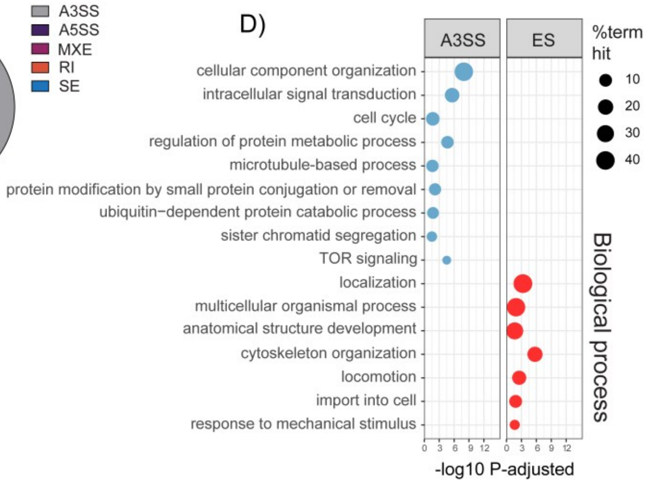


E)

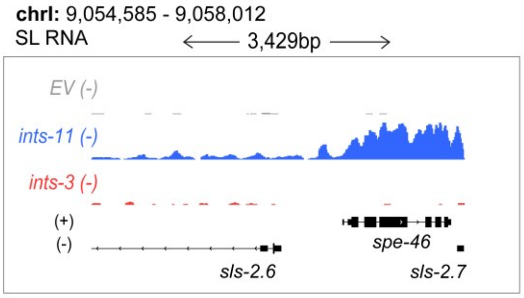


F)

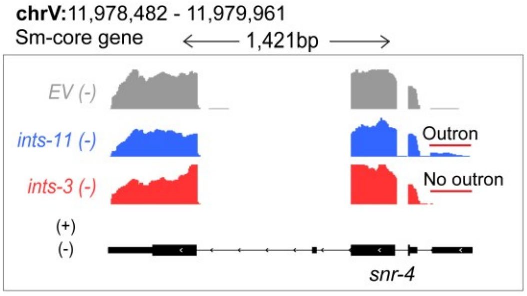


G)

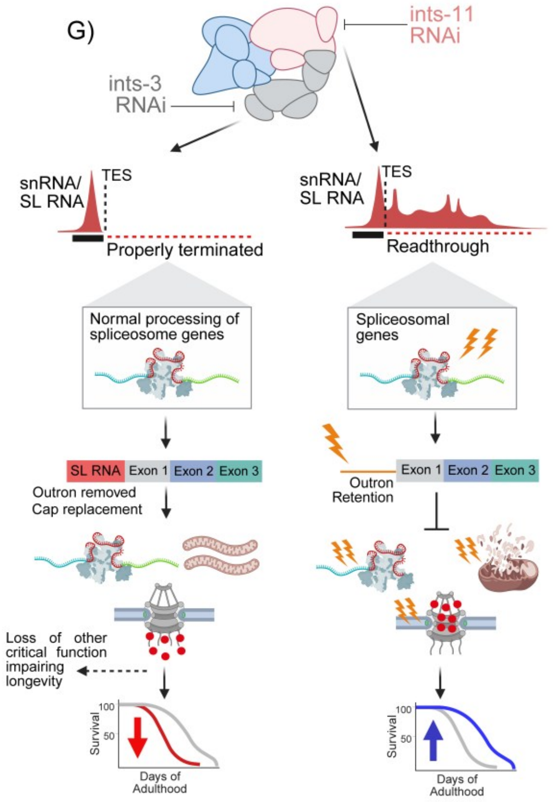

bioRxiv preprint doi: https://doi.org/10.64898/2026.04.18.719358; this version posted April 21, 2026. The copyright holder for this preprint (which was not certified by peer review) is the author/funder, who has granted bioRxiv a license to display the preprint in perpetuity. It is made available under aCC-BY 4.0 International license.

**Supplemental figure 4. A)** Volcano plots representing percentage spliced in (PSI) for alternatively spliced isoforms identified within RNA-seq datasets. **B)** Summary table for differential splicing events. **C)** Pie chart summarising distribution of alternative splicing events across splicing classes. **D)** Gene ontology enrichment for alternatively spliced events. **E)** Genome coverage tracks highlighting lack of SL RNA termination defects from *ints-3* RNAi (negatively effects lifespan), but stark readthrough from *ints-11* RNAi (lifespan extending effects). **F)** Genome coverage tracks highlighting the presence/ absence of outron retention from *ints-11* versus *ints-3* RNAi, respectively. **G)** Model indicating defective termination of sn/SL RNA and consequential impacts on spliceosomal genes sit upstream of other contributing longevity programmes from loss of *ints-11*.

bioRxiv preprint doi: https://doi.org/10.64898/2026.04.18.719358; this version posted April 21, 2026. The copyright holder for this preprint (which was not certified by peer review) is the author/funder, who has granted bioRxiv a license to display the preprint in perpetuity. It is made available under aCC-BY 4.0 International license.

**Supplemental figure 5.**

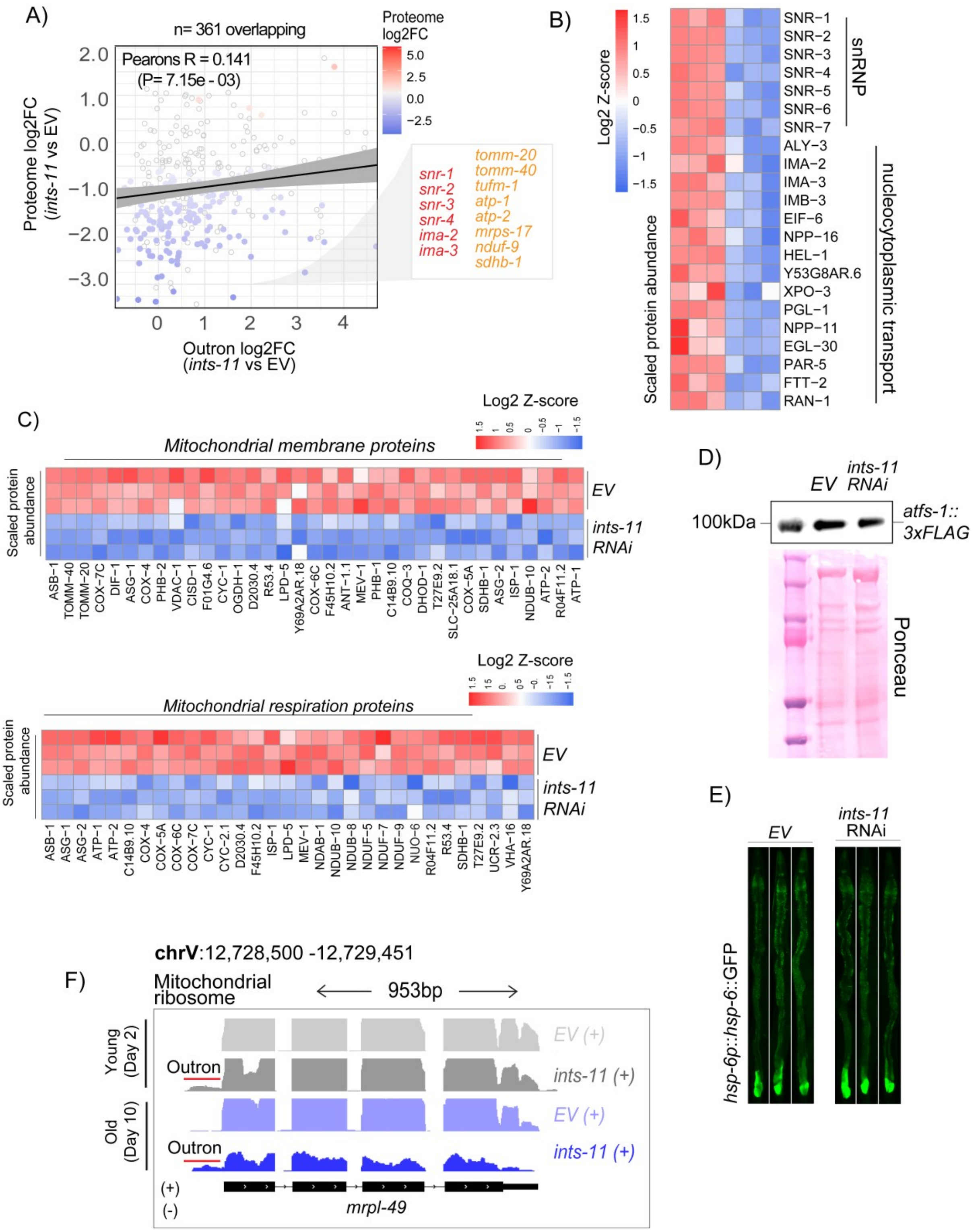

bioRxiv preprint doi: https://doi.org/10.64898/2026.04.18.719358; this version posted April 21, 2026. The copyright holder for this preprint (which was not certified by peer review) is the author/funder, who has granted bioRxiv a license to display the preprint in perpetuity. It is made available under aCC-BY 4.0 International license.

**Supplemental figure 5. A)** Correlation plot comparing outron-retaining genes with protein abundance levels from whole-animal proteomics. **B)** Log2 Z-score scaled heatmaps of core spliceosomal and nucleocytoplasmic proteins implicated in trans-splicing and snRNP biogenesis pathways. **C)** Heatmaps of mitochondrial outer-membrane (top) and respiratory (bottom) proteins. **D)** Western blot of an endogenously-tagged ATFS-1 strain after 5-days of *ints-11* RNAi, with ponceau staining as a representative loading control. **E)** Representative microscopy images of the *phsp-6::hsp-6::GFP* reporter strain after 5-days of *ints-11* RNAi. **F)** Genome browser tracks of a mitochondrial ribosome gene exhibiting outron retention in day-2 and day-10 *ints-11* silenced animals.
